# *Artemia salina* as a model organism in toxicity assessment of nanoparticles

**DOI:** 10.1186/s40199-015-0105-x

**Published:** 2015-02-24

**Authors:** Somayeh Rajabi, Ali Ramazani, Mehrdad Hamidi, Tahereh Naji

**Affiliations:** Cell and Molecular Biology Departments, Pharmaceutical Sciences Branch, Islamic Azad University, Tehran, Iran; Biotechnology Departments, School of Pharmacy, Zanjan University of Medical Sciences, Zanjan, Iran; Zanjan Pharmaceutical Nanotechnology Research Center, Zanjan University of Medical Sciences, Zanjan, Iran

**Keywords:** *Artemia salina*, Toxicity, Nanoparticle, Cell culture

## Abstract

**Background:**

Because of expanding presence of nanomaterials, there has been an increase in the exposure of humans to nanoparticles that is why nanotoxicology studies are important. A number of studies on the effects of nanomatrials in *in vitro* and *in vivo* systems have been published. Currently cytotoxicity of different nanoparticles is assessed using the 3-(4,5-dimethylthiazol-2-yl)-2,5-diphenyltetrazolium bromide (MTT) assay on different cell lines to determine cell viability, a tedious and expensive method. The aim of this study was to evaluate the *Artemia salina* test in comparison with the MTT assay in the assessment of cytotoxicity of nanostructures because the former method is more rapid and convenient and less expensive.

**Methods:**

At the first stage, toxicity of different nanoparticles with different concentrations (1.56–400 μg/mL) was measured by means of the brine shrimp lethality test. At the second stage, the effect of nanoparticles on the viability of the L929 cell line was assessed using the MTT assay. Experiments were conducted with each concentration in triplicate.

**Results:**

The results obtained from both tests (*A. salina* test and MTT assay) did not have statistically significant differences (*P* > 0.05).

**Conclusions:**

These findings suggest that the *A. salina* test may expedite toxicity experiments and decrease costs, and therefore, may be considered an alternative to the *in vitro* cell culture assay.

## Background

Nanoscience is a novel science that is being developed to probe and manipulate matter on the scale of single atoms and molecules. Physicist Richard P. Feynman was the first to mention molecular machines built with atomic precision at a meeting of the American Physical Society in 1959 [1], and Noro Taniguchi, a professor at the University of Tokyo, coined the term nanotechnology in 1979 [2]. Nanotechnology is the use of nanoscience to design NMs (nanomaterials) and NPs (nanoparticles), with structural components between 1 and 100 nanometers; it is thought to be one of the key technologies of the 21st century [3,4]. In the above range, physicochemical characteristics of NPs in biological systems can vary. Some potential hazards have been identified in the life cycle of NMs and NPs [5,6]. Growing research and development in nanotechnology have resulted in the identification of many unique properties of nanomaterials such as enhanced magnetic, catalytic, optical, electrical, and mechanical properties when compared to conventional formulations of the same materials [7]. While nanoparticles have a wide variety of functions, there has been increasing issues and debate amongst the regulatory and scientific community regarding the fate of nanoparticles in biological systems and associated side effects these agents might have on living organisms [8-12]. These materials are increasingly used for commercial purposes and leading to direct and indirect exposure of humans [13]. Any *in vivo* use of nanoparticles requires thorough understanding of the kinetics and toxicology of the particles, establishment of principles and test procedures to ensure safe manufacture and usage of nanomaterials, and comprehensive training of personnel in safety and potential hazards of nanotechnology [13]. Nanotoxicology research is applied to various fields including biology and pathology, but typically to pharmacology and to the use of NMs and nanodevices for diagnostic and therapeutic purposes. Therefore, a key goal for toxicologists is to identify *in vitro* and *in vivo* assays accurately reflecting the ability of NPs to induce toxic effects in the humans and in the environment. In addition, standardized tests for both *in vitro* and *in vivo* studies are needed to develop better and more rapid screening techniques and to predict toxicity [14,15]. The cytotoxicity effects of NPs were investigated in a multitude of animal models by means of *in vivo* tests employing the typical NP exposure routes, i.e., pulmonary, oral, dermal, and injection based [16]. The cost and labor intensiveness of the *in vivo* studies have led researchers to the use of *in vitro* methods for assessment of NPs cytotoxicity. In addition, animal rights advocates have criticized the use of animals in nanotechnology experiments. All *in vivo* studies must be conducted with the approval of regulatory bodies such as IACUC (an Institutional Animal Care and Use Committee) to ensure ethical treatment of animals [16]. For the above reasons, *in vitro* techniques are increasingly used for the analysis of cytotoxicity of NPs including cell culture, the WST-1 assay [17,18], XTT assay, MTT assay [19,20], LDH assay, BrdU assay, and fluorescence microscopy [21,22]. Currently, cytotoxicity testing of various NPs in cell culture involves the MTT assay, which determines cell viability based on mitochondrial function by measuring the activity of mitochondrial enzymes [23-27]. In this test, tetrazolium is reduced by mitochondrial succinate dehydrogenase of live cells to water-insoluble purple formazan crystals, which are subsequently solubilized using an organic solvent (e.g., dimethyl sulfoxide; DMSO) [28]. The cell viability is quantified based on absorbance of the solution at 570 nm. Therefore, the MTT assay requires solubilization steps with tetrazolium, which is toxic to cells and can interfere with some chemical reactions [28]. The cytotoxicity assays are often tedious and expensive, and there is a lack of a simple and rapid screening procedure. Nowadays, brine shrimp lethality assays are extensively used in research and applied toxicology [29]. There is a tendency to use an *Artemia salina* assay in toxicological tests that screen a large number of extracts for drug discovery in medicinal plants [30-33]. This is because in this case, aseptic techniques are not required, and thus *A. salina* assays could replace the more ethically challenging MTT assay that requires animal serum [34]. This assay was proposed by Michael and coworkers in 1959 and was later adopted by many laboratories as a method for preliminary estimation of toxicity [35]. *Artemia* is one of the most valuable test organisms available for ecotoxicity testing, and the available research suggests that several applications of *Artemia* to toxicology and ecotoxicology will continue to be used widely [36]. Because of the rapidity, convenience, and low cost of *Artemia*-based assays, we decided to evaluate the *A. salina* test in comparison with the MTT assay in the assessment of cytotoxicity of different classes of NPs.

## Materials and methods

### Materials

Fetal bovine serum (FBS), phosphate-buffered saline (PBS), trypsin, penicillin, streptomycin, DMSO, 3-[4,5-dimethylthiazol-2-yl]-2,5-diphenyl tetrazolium bromide (MTT), Triton X-100, and the RPMI-1640 medium supplemented with 10% heat inactivated FBS were purchased from Sigma–Aldrich. The mouse fibroblast cell line (L929) was provided by Pasteur Institute of Iran.

### Synthesis of NPs

Sixteen NPs from different classes (Table 1) were prepared by the Nanotechnology Laboratory of School of Pharmacy of Zanjan University of Medical Sciences. The zeta potential and particle size distribution of the prepared nanoparticles were determined by photon correlation spectroscopy (PCS) using a Nano/zetasizer (Malvern Instruments, Nano ZS, Worcestershire, UK) working on the dynamic light scattering (DLS) platform.

**Table 1:** **Names and characteristics of NPs used in this study**

**NPs class**	**NPs name**	**Size NPs (nm)**	**Zeta potential (mV)**
Inorganic nanoparticles	Magnetic	55	- 31
	Nanosfer	97	+8.9
Lipid-base nanoparticles	Liposome	139.3	- 28
	Coated SLNs	464.6	+20
	Uncoated SLNs	176.3	- 45
	Nanogele + SLN	376.6	+4
Polymeric nanoparticles	Nanogele	270	+22
	Micellar	97.9	- 1.10
	PAMAM (G5)	6	+36.8
	PAMAM-FA	55	+36.4
	PAMAM-PEG-FA	70	9.12
Drug nanoparticles	Nanosuspansion Atorvastatin	269.8	ND
	Nanosuspansion Ibuprofen	160.9	ND
	Nanosuspansion Repaglinide	260.6	ND
	Nanosuspansion Cyclosporin	220.5	ND
	Nanosuspansion Azitromycin	270	ND

ND; not determined.

### Cell culture and determination of cytotoxicity by MTT assay

The effect of NPs on the viability of L929 cells was assessed by means of the MTT assay. After thawing, the cells were cultured in the RPMI 1640 medium containing 10% FBS, penicillin (100 units/mL), and streptomycin (100 mg/mL) at 37°C in a humidified 5% CO_2_ incubator. The cells were seeded in a 96-well plate at a density of 5,000 cells per well (the cells were stained with trypan blue and counted with haemocytometer). These cells were incubated overnight at 37°C before the cell viability test. A stock suspension of each NP at 50 mg/mL in distilled water was prepared. After that, fresh suspensions of different concentrations of NPs (two fold serial dilutions from 1.56–400 μg/mL) were made using serial dilution of the stock suspensions of NPs in the RPMI 1640 medium, immediately before use. We added 200 μL of a suspension (different concentrations of NPs) to each well of the microtiter plates. The cells were incubated for 24 h under the same conditions. Wells without any NPs served as a negative control. The experiments were performed in triplicate for each concentration. To assess cell survival, 100 μL of an MTT solution (2 mg/mL in PBS) was added to each well and incubated for 3 h at 37°C to produce insoluble formazan. Then, 100 μL of DMSO was added to dissolve formazan crystals, and the absorbance was measured on an Infinite M200 microplate reader (Tecan) at 570 nm, with 630 nm as a reference wavelength. The percentage of cell viability was calculated using the formula (A_test_/A_control_) × 100, where A_test_ is the mean absorbance of treated cells and A_control_ is the mean absorbance of a negative control.

### Toxicity testing by *A. salina*

*A. salina* eggs were purchased from the Aquatic Animal Research Center, Urmia University, Urmia, Iran. Dried cysts were placed in a bottle containing artificial sea water which was prepared by dissolving 35 g of sodium chloride in 1 L of distilled water. After 36–48 h incubation at room temperature (28–30°C) under conditions of strong aeration and continuous illuminations [33], the larvae (nauplii) hatched within 48 h.

The evaluation of cytotoxicity of NPs in *A. salina* was performed according to the previous methods [30,33,37,38]. The assay was carried out on larvae of brine shrimp (*A. salina* Leach.). A stock solution of 50 mg of nanoparticles in 1 mL of distilled water was prepared. Then, fresh suspensions with different concentrations of NPs (two fold serial dilutions from 1.56–400 μg/mL) were made by means of serial dilution of the stock suspensions of NPs in artificial sea water (35 g/L) immediately before use. We added 200 μL of a suspension (different concentrations of NPs) to each well of the 96-well microtiter plates. After that, 10 nauplii per well were added in the 96-well plates and incubated at room temperature for 24 h. The numbers of surviving nauplii in each well were counted under a stereoscopic microscope after 24 h. The experiments were conducted in triplicate for each concentration. The negative control wells contained 10 nauplii and artificial sea water only. The percentages of deaths were calculated by comparing the number of survivors in the test and control wells. The lethality was calculated using Abbott’s formula as follows:% Lethality = [(Test − Control)/Control] × 100.

### Statistical analysis

All experiments were done in triplicate and the results were calculated as a mean ± standard deviation (SD). The experimental data were processed using the paired sample *t*-test, Pearson correlation and linear regression analysis of the SPSS version 16.0 software for Windows. The toxicity of each nanoparticle was calculated from the 50% lethality dose (LD_50_) by means of Finney’s Probit analysis [39].

## Results

### Cytotoxicity of nanostructures by the MTT assay

The MTT assay is a viable method for assessing *in vitro* cytotoxicity of NPs. In this study, L929 cells were treated with different concentrations (0.78–200 μg/mL) of the 16 NPs (Table 1). Cell viability was determined 24 hours after the treatment. The results are presented in Table 2. Uncoated solid lipid nanoparticles (SLNs), Nanogel + SLN, Bare Nanogel, polyamidoamine (PAMAM; G5), and PAMAM-FA demonstrated moderate cytotoxicity. In contrast, the NPs with IC_50_ > 200 μg/mL were not toxic to the L929 cell line. The cytotoxicity was weak when the IC_50_ values were between 150 and 200 μg/mL.

**Table 2:** **NPs toxicity assay by** ***Artemia salina*** **and MTT assay**

**NPs name**	***Artemia salina*** **assay, LC**_**50**_ **(μg/m)**	**95% confidence limits for concentration**		**MTT assay, IC**_**50**_ **(μg/ml)**	**95% confidence limits for concentration**	
		**Lower bound**	**Upper bound**		**Lower bound**	**Upper bound**
Magnetic	698.710	431.764	2669.870	997.402	527.931	3340.601
Nanosfer	302.001	215.853	497.009	207.431	141.862	365.065
Liposome	751.249	432.851	6492.955	1002.666	543.931	4358.603
Coated SLNs	360.594	285.519	501.000	605.594	472.900	1248.021
Uncoated SLNs	239.040	192.979	310.917	149.018	90.886	328.486
Nanogele + SLN	19.656	15.141	24.908	113.903	58.153	380.573
Nanogele	40.440	8.221	95.196	130.171	77.672	285.277
Micellar	560.060	352.592	1801.667	686.002	263.396	1329.851
PAMAM (G5)	145.6	60.435	671.764	72.254	41.149	139.077
PAMAM-FA	213.316	185.467	839.316	99.783	61.360	130.925
PAMAM-PEG-FA	401.21	256.482	1002.310	270.585	129.042	496.556
Nanosuspansion Atorvastatin	417.349	272.658	1046.615	807.668	301.840	1080.114
Nanosuspansion Ibuprofen	373.526	285.877	559.357	156.433	117.536	244.043
Nanosuspansion Repaglinide	807.754	488.424	4335.993	297.756	221.995	527.574
Nanosuspansion Cyclosporin	531.961	368.036	1095.239	167.965	108.493	230.459
Nanosuspansion Azitromycin	110.316	57.555	231.560	162.512	168.114	249.652

### Cytotoxicity of nanostructures in the brine shrimp assay

**Figure 1:**
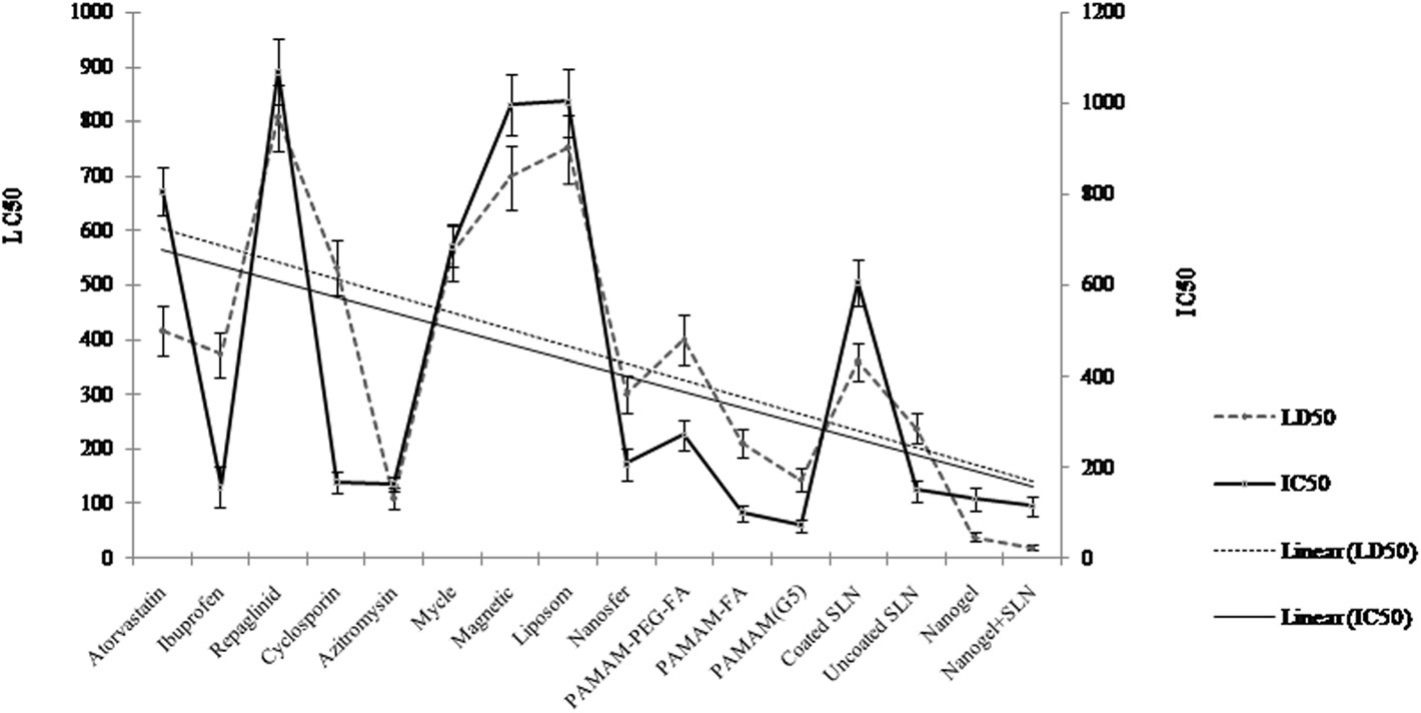
**Comparison of** ***Artemia salina*** **and MTT assay results**

The brine shrimp lethality assay was also used to determine the cytotoxicity of NPs. According to the results (Table 2), of the 16 NPs that we screened for lethality in *A. salina*, only two NPs (Nanogel + SLN and Nanogel) showed strong toxicity (LC_50_ < 100 μg/mL). In contrast, NPs of Uncoated SLN, PAMAM (G5), Nanosuspension Ibuprofen, and Nanosuspension Azithromycin exhibited moderate cytotoxicity (LC_50_ ranged between 100 and 500 μg/mL), and the other NPs showed weak cytotoxicity in *A. salina* (LC_50_ range 500–1000 μg/mL) [37,40]. Comparison between the results of two methods is indicated in Figure 1. As shown in Figure 1, the trend lines and the direction of the graphs are in same direction.

## Discussion

Several assays for eco-toxicological testing of nanomaterials have been developed. Different model systems such as bacteria [41], fathead minnows [42], zebrafish embryos [43], copepod [44], Daphnia [45,46], and rainbow trout [47,48] have been reported [49]. In addition to standard tests, there is a need to establish better, rapid and convenient methods to predict the toxic effects of nanomaterials. Till now, A few studies have reported the toxicity effect of nanomaterials on *A. salina* [38,50-52]. These studies were investigated on metal nanoparticles and we want develop the *A. salina* assay for toxicity assessment of different class of nanomaterials especially those used for drug deliveries.

In this work, cytotoxicity of 16 NPs was assessed using two methods: the brine shrimp lethality assay and the MTT assay in L929 cells. According to the results (Figure 1) the correlation between the LC_50_ and IC_50_ values is significant (R^2^ = 0.72, *P* = 0.000). This mean that 72% variability noted in MTT method could be accounted for by brine shrimp lethality assay and 28% is unaccounted for due to measurement error. There was not statistically significant difference between two assays when the results were compared with paired t test (*P* = 0.402). Also the comparison between LC_50_ and IC_50_ mean values statistically analyzed by chi square test and the result showed that there is no differences between two assay methods (P = 0.235). This mean that results obtained by brine shrimp lethality assay is comparable with MTT results. Both the ranking and the degree of cytotoxicity were similar between the brine shrimp lethality assay and the MTT assay. As it has been shown in Table 2, the ranges of 95% confidence limit are wide. The width of the confidence interval for an individual study depends to a large extent on the sample size. Larger studies tend to give more precise estimates of effects (and hence have narrower confidence intervals) than smaller studies. In order to obtain a more reliable estimate of the confidence interval it may be necessary to perform several independent assays and to combine these into one single confidence interval [53]. The results demonstrate the ability of the brine shrimp lethality assay to accurately quantify cytotoxicity of NPs and to replace the MTT assay, which is expensive and tedious. In this field, cytotoxicity assays and experimental procedures often lack a simple, convenient, and rapid screening method. On the other hand, the brine shrimp lethality assay has been used in toxicology research routinely for over thirty years [36]. The genus *Artemia* has a several advantages that make it ideal for general toxicity assays including wide geographical distribution, adaptability to extreme conditions, capability to use several nutrient resources, and availability of their cysts for collection [36]. The brine shrimp assay is convenient because it is rapid (24 h), economical, and simple. The eggs of *A. salina* are readily available at low cost and remain viable for years in dry storage. The assay easily accommodates a large number of nauplii for statistical validation and no special equipment is needed. Moreover, this assay does not require animal serum and thereby it prevents unnecessary use of animals in scientific experiments. In summary, it is possible to measure cytotoxicity of NPs using the brine shrimp lethality assay instead of the common *in vitro* cell culture assays.

## Conclusion

This work shows that the brine shrimp lethality assay can be used to study toxicity of nanostructures. Self-sufficiency and rapid results are important advantages of this method. *Artemia*-based toxicity assay of NPs are cheap, continuously available, simple and reliable and are thus an important answer to routine needs of toxicity screening, for industrial monitoring requirements or for regulatory purposes. Our data are expected to facilitate pharmacological and nanotoxicological research.
